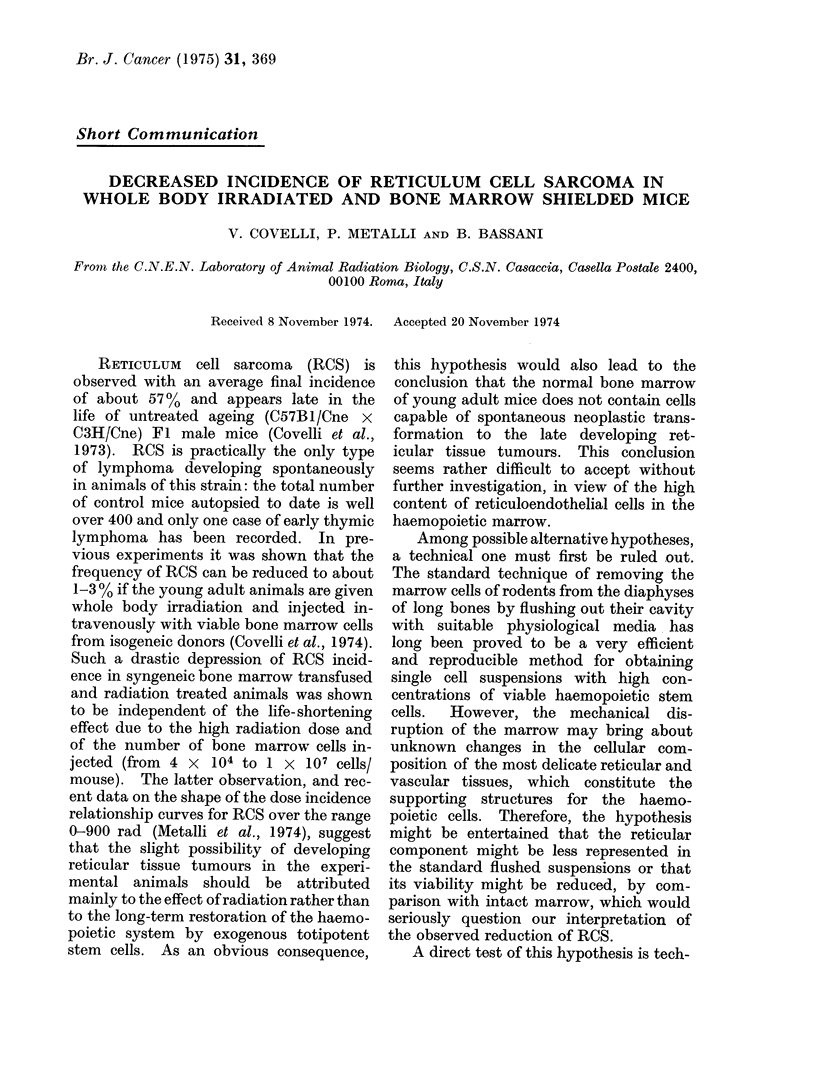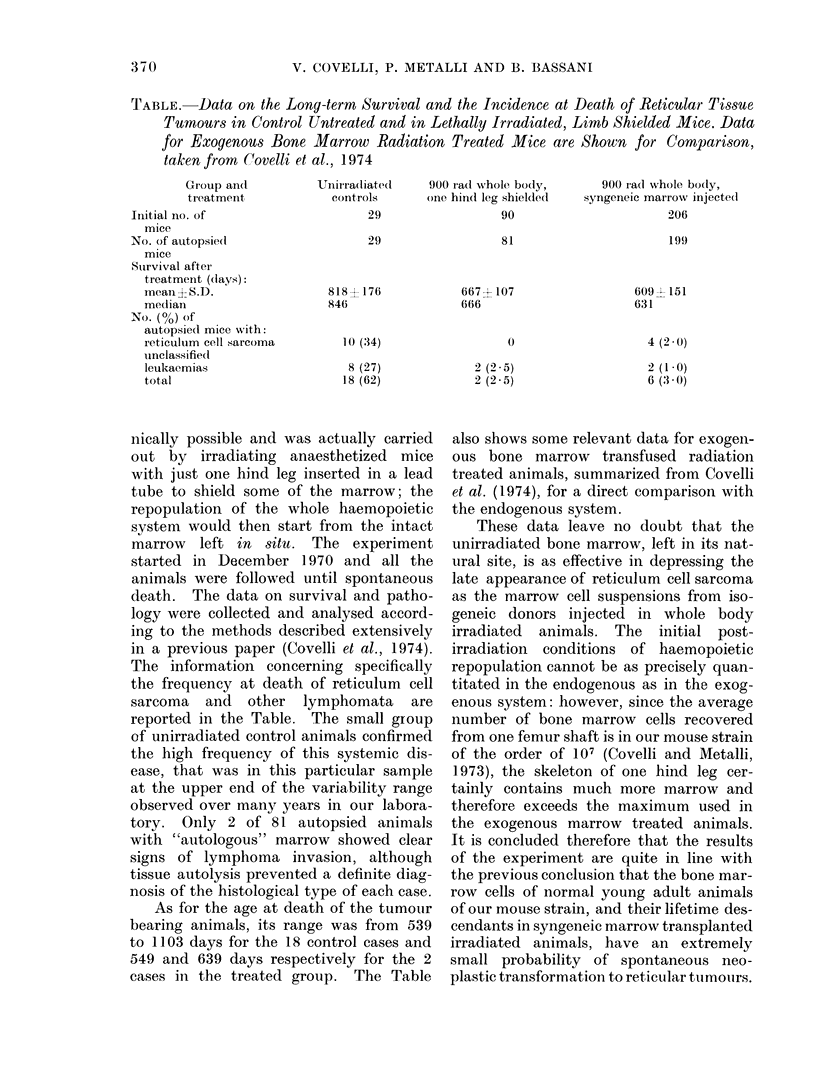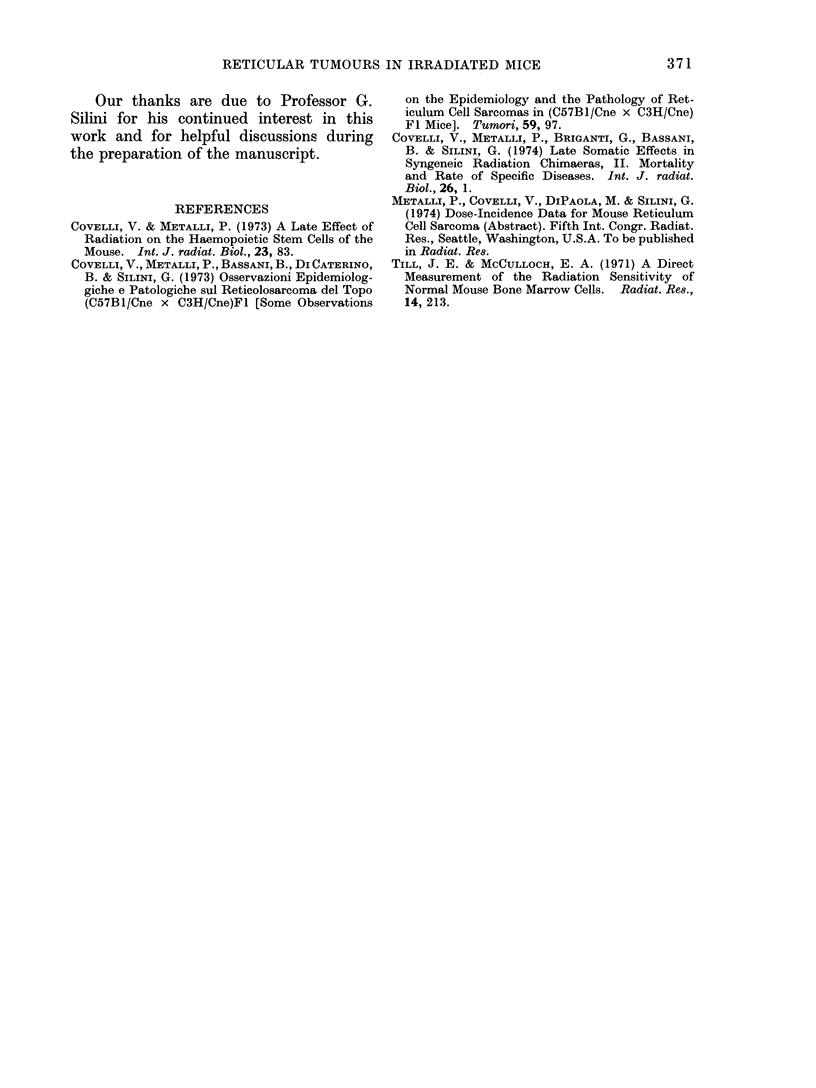# Decreased incidence of reticulum cell sarcoma in whole body irradiated and bone marrow shielded mice.

**DOI:** 10.1038/bjc.1975.73

**Published:** 1975-03

**Authors:** V. Covelli, P. Metalli, B. Bassani


*Br. J. Cancer* (1975) **31**, 369

***Short Communication***

### DECREASED INCIDENCE OF RETICULUM CELL SARCOMA IN WHOLE BODY IRRADIATED AND BONE MARROW SHIELDED MICE

V. COVELLI, P. METALLI AND B. BASSANI

*From the C.N.E.N. Laboratory of Animal Radiation Biology, C.S.N. Casaccia, Casella Postale* 2400, 00100 *Roma, Italy*

Received 8 November 1974. Accepted 20 November 1974

RETICULUM cell sarcoma (RCS) is observed with an average final incidence of about 57% and appears late in the life of untreated ageing (C57Bl/Cne × C3H/Cne) F1 male mice (Covelli *et al.*, 1973). RCS is practically the only type of lymphoma developing spontaneously in animals of this strain: the total number of control mice autopsied to date is well over 400 and only one case of early thymic lymphoma has been recorded. In previous experiments it was shown that the frequency of RCS can be reduced to about 1–3% if the young adult animals are given whole body irradiation and injected intravenously with viable bone marrow cells from isogeneic donors (Covelli *et al.*, 1974). Such a drastic depression of RCS incidence in syngeneic bone marrow transfused and radiation treated animals was shown to be independent of the life-shortening effect due to the high radiation dose and of the number of bone marrow cells injected (from $4 \times 10^4$ to $1 \times 10^7$ cells/mouse). The latter observation, and recent data on the shape of the dose incidence relationship curves for RCS over the range 0–900 rad (Metalli *et al.*, 1974), suggest that the slight possibility of developing reticular tissue tumours in the experimental animals should be attributed mainly to the effect of radiation rather than to the long-term restoration of the haemopoietic system by exogenous totipotent stem cells. As an obvious consequence, this hypothesis would also lead to the conclusion that the normal bone marrow of young adult mice does not contain cells capable of spontaneous neoplastic transformation to the late developing reticular tissue tumours. This conclusion seems rather difficult to accept without further investigation, in view of the high content of reticuloendothelial cells in the haemopoietic marrow.

Among possible alternative hypotheses, a technical one must first be ruled out. The standard technique of removing the marrow cells of rodents from the diaphyses of long bones by flushing out their cavity with suitable physiological media has long been proved to be a very efficient and reproducible method for obtaining single cell suspensions with high concentrations of viable haemopoietic stem cells. However, the mechanical disruption of the marrow may bring about unknown changes in the cellular composition of the most delicate reticular and vascular tissues, which constitute the supporting structures for the haemopoietic cells. Therefore, the hypothesis might be entertained that the reticular component might be less represented in the standard flushed suspensions or that its viability might be reduced, by comparison with intact marrow, which would seriously question our interpretation of the observed reduction of RCS.

A direct test of this hypothesis is tech-

TABLE.—*Data on the Long-term Survival and the Incidence at Death of Reticular Tissue Tumours in Control Untreated and in Lethally Irradiated, Limb Shielded Mice. Data for Exogenous Bone Marrow Radiation Treated Mice are Shown for Comparison, taken from Covelli et al.*, 1974

| Group and treatment | Unirradiated controls | 900 rad whole body, one hind leg shielded | 900 rad whole body, syngeneic marrow injected |
|---|---|---|---|
| Initial no. of mice | 29 | 90 | 206 |
| No. of autopsied mice | 29 | 81 | 199 |
| Survival after treatment (days): | | | |
| mean ± S.D. | 818 ± 176 | 667 ± 107 | 609 ± 151 |
| median | 846 | 666 | 631 |
| No. (%) of autopsied mice with: | | | |
| reticulum cell sarcoma | 10 (34) | 0 | 4 (2·0) |
| unclassified leukaemias | 8 (27) | 2 (2·5) | 2 (1·0) |
| total | 18 (62) | 2 (2·5) | 6 (3·0) |

nically possible and was actually carried out by irradiating anaesthetized mice with just one hind leg inserted in a lead tube to shield some of the marrow; the repopulation of the whole haemopoietic system would then start from the intact marrow left *in situ*. The experiment started in December 1970 and all the animals were followed until spontaneous death. The data on survival and pathology were collected and analysed according to the methods described extensively in a previous paper (Covelli *et al.*, 1974). The information concerning specifically the frequency at death of reticulum cell sarcoma and other lymphomata are reported in the Table. The small group of unirradiated control animals confirmed the high frequency of this systemic disease, that was in this particular sample at the upper end of the variability range observed over many years in our laboratory. Only 2 of 81 autopsied animals with "autologous" marrow showed clear signs of lymphoma invasion, although tissue autolysis prevented a definite diagnosis of the histological type of each case.

As for the age at death of the tumour bearing animals, its range was from 539 to 1103 days for the 18 control cases and 549 and 639 days respectively for the 2 cases in the treated group. The Table also shows some relevant data for exogenous bone marrow transfused radiation treated animals, summarized from Covelli *et al.* (1974), for a direct comparison with the endogenous system.

These data leave no doubt that the unirradiated bone marrow, left in its natural site, is as effective in depressing the late appearance of reticulum cell sarcoma as the marrow cell suspensions from isogeneic donors injected in whole body irradiated animals. The initial post-irradiation conditions of haemopoietic repopulation cannot be as precisely quantitated in the endogenous as in the exogenous system: however, since the average number of bone marrow cells recovered from one femur shaft is in our mouse strain of the order of $10^7$ (Covelli and Metalli, 1973), the skeleton of one hind leg certainly contains much more marrow and therefore exceeds the maximum used in the exogenous marrow treated animals. It is concluded therefore that the results of the experiment are quite in line with the previous conclusion that the bone marrow cells of normal young adult animals of our mouse strain, and their lifetime descendants in syngeneic marrow transplanted irradiated animals, have an extremely small probability of spontaneous neoplastic transformation to reticular tumours.

Our thanks are due to Professor G. Silini for his continued interest in this work and for helpful discussions during the preparation of the manuscript.

### REFERENCES

COVELLI, V. & METALLI, P. (1973) A Late Effect of Radiation on the Haemopoietic Stem Cells of the Mouse. *Int. J. radiat. Biol.*, **23**, 83.

COVELLI, V., METALLI, P., BASSANI, B., DI CATERINO, B. & SILINI, G. (1973) Osservazioni Epidemiologiche e Patologiche sul Reticolosarcoma del Topo (C57B1/Cne × C3H/Cne)F1 [Some Observations on the Epidemiology and the Pathology of Reticulum Cell Sarcomas in (C57B1/Cne × C3H/Cne) F1 Mice]. *Tumori*, **59**, 97.

COVELLI, V., METALLI, P., BRIGANTI, G., BASSANI, B. & SILINI, G. (1974) Late Somatic Effects in Syngeneic Radiation Chimaeras, II. Mortality and Rate of Specific Diseases. *Int. J. radiat. Biol.*, **26**, 1.

METALLI, P., COVELLI, V., DIPAOLA, M. & SILINI, G. (1974) Dose-Incidence Data for Mouse Reticulum Cell Sarcoma (Abstract). Fifth Int. Congr. Radiat. Res., Seattle, Washington, U.S.A. To be published in *Radiat. Res.*

TILL, J. E. & MCCULLOCH, E. A. (1971) A Direct Measurement of the Radiation Sensitivity of Normal Mouse Bone Marrow Cells. *Radiat. Res.*, **14**, 213.